# Supplementary material for: High-performance Kerr microresonator optical parametric oscillator on a silicon chip
Source: Nat Commun. 2023 Jan 16;14:242. doi: 10.1038/s41467-022-35746-9 (PMC9842726; doi:10.1038/s41467-022-35746-9)
Supplement: Supplementary file 1 — Supplementary Information [file 41467_2022_35746_MOESM1_ESM.pdf]

Supplementary Material

This supplementary material contains figures and tables that illustrate the experimental setup and describe the losses in our experiment, and were referenced in the main text and methods sections.

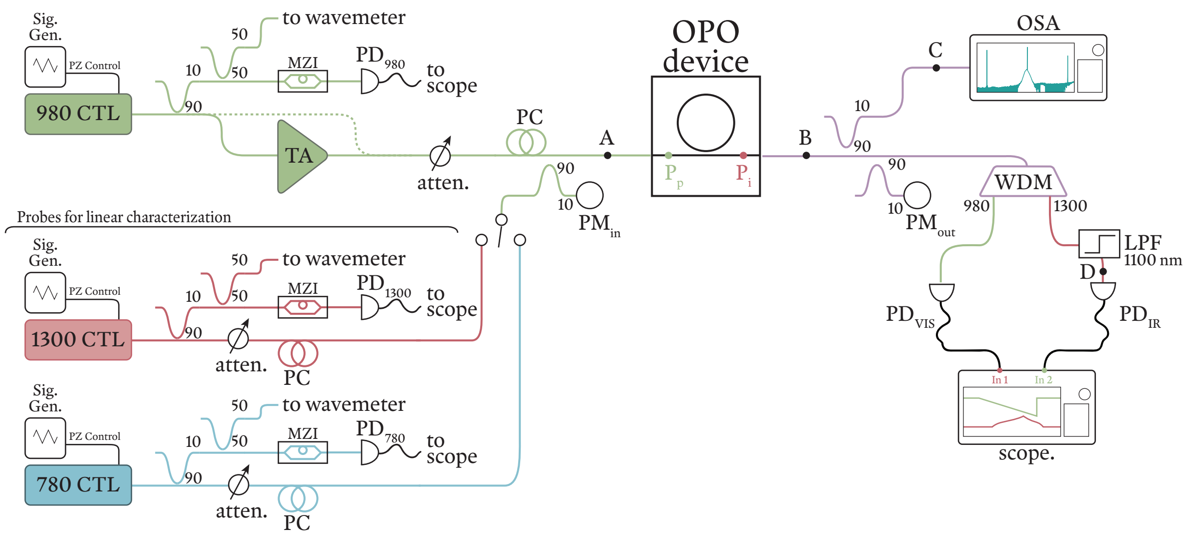

Supplementary Fig. S1. **OPO characterization setup.** The OPO devices are pumped through a lensed optical fiber with an amplified 980 nm continuously tunable laser (CTL), and the out-coupled OPO output (also via a lensed optical fiber) is fed through a wavelength division multiplexer (WDM) to separate the pump and idler channels or wavelength-resolved using on an optical spectrum analyzer (OSA). A lowpass filter (LPF) is used to attenuate all remaining pump light in the idler channel. Afterwards, photodetectors PD_{VIS} and PD_{IR} respectively record the pump power P_p and idler power P_i on chip, as indicated by the green (P_p) and red (P_i) labels in the OPO device, by using transmission calibrations summarized in Table S1. For characterization of frequency mismatch and cavity Q in the signal and idler bands, additional CTL sources in the relevant wavelength bands are used. Each CTL is connected to a Mach–Zehnder interferometer (MZI) and a wavemeter for accurate frequency monitoring. CTL sources are connected to alternators (atten.) and polarization controllers (PC). Power meters PM_{in} and PM_{out} monitor the insertion losses to the chip.

Path	Wavelength (nm)	Loss (dB)
A → B	980	4.5
	1300	3.7
B → C	980	0.5
	1300	0.7
B → D	1300	5.5

Supplementary Tab. S1. **Typical system losses.** Typical values for losses through various paths in the system. Path labels correspond to the labeled points in Supplementary Fig. S1. These losses account for offsets in the power values reported in OSA traces (Fig. 3 and Fig. 4) relative to their y-axes.

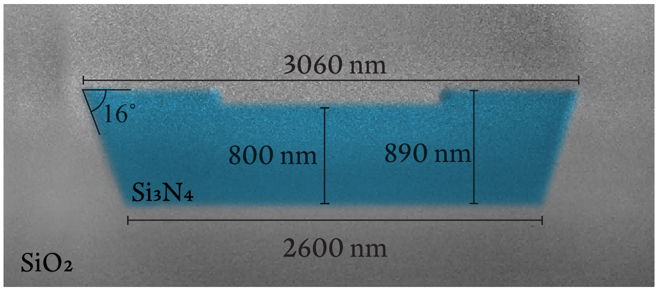

Supplementary Fig. S2. **Geometric characterization of resonator.** A representative cross section of the resonators used in this study, acquired by focused ion beam milling. The blue-shaded region depicts a Si_3N_4 ring resonator with an upside-down trapezoidal geometry as described in the text, with typical dimensions included. The notch in the top portion of the device is likely due to a combination of the reflow and chemical mechanical polishing processes used in fabrication, but has limited impact on our study.
